# Fabrication of Phosphate-Imprinted PNIPAM/SiO_2_ Hybrid Particles and Their Phosphate Binding Property

**DOI:** 10.3390/polym11020253

**Published:** 2019-02-02

**Authors:** Zheng Cao, Yuyuan Chen, Dan Li, Junfeng Cheng, Chunlin Liu

**Affiliations:** 1 Jiangsu Key Laboratory of Environmentally Friendly Polymeric Materials, School of Materials Science and Engineering, Jiangsu Collaborative Innovation Center of Photovoltaic Science and Engineering, Changzhou University, Changzhou 213164, China; zcao@cczu.edu.cn (Z.C.); 15189781265@163.com (Y.C.); 15151992892@163.com (D.L.); 2 Key Laboratory of Synthetic and Self-Assembly Chemistry for Organic Functional Molecules, Shanghai Institute of Organic Chemistry, Chinese Academy of Sciences, 345 Lingling Road, Shanghai 200032, China; 3 National Experimental Demonstration Center for Materials Science and Engineering (Changzhou University), Changzhou 213164, China; 4 Huaide College, Changzhou University, Changzhou 213016, China

**Keywords:** *N*-isopropylacrylamide, molecular imprinting, polymer/inorganic, phosphate, binding

## Abstract

A SiO_2_ microsphere imprinted by phosphate ions was prepared with the use of phosphate ion as the template molecule and tetraethoxysilane as the precursor. Thereafter, the imprinted SiO_2_ microspheres were modified with 3-(trimethoxysilyl)propyl methacrylate (TMSPMA@SiO_2_), followed by introducing the double bond. In the presence of TMSPMA@SiO_2_, using *N*-isopropylacrylamide as monomer, and potassium persulfate as initiator, polymer/inorganic hybrid particles (PNIPAM/SiO_2_) were prepared. Fourier transform infrared spectroscopy, thermogravimetric analysis, nitrogen adsorption-desorption test, and transmission electron microscope were employed for the characterization of molecular imprinted SiO_2_ microspheres and PNIPAM/SiO_2_ hybrid particles. The effects of phosphate concentration, pH value, and adsorption temperature on the phosphate binding properties of PNIPAM/SiO_2_ hybrid particles were studied by UV-vis spectrophotometer. The experimental results shed light on the fact that the PNIPAM structure is beneficial for the improvement of the adsorption ability of phosphate-imprinted SiO_2_ microspheres. With the increase in the initial phosphate concentration, the adsorption capacity of hybrid particles to phosphate ions increased to 274 mg/g at pH = 7 and 15 °C. The acid condition and the temperature below the low critical solution temperature (LCST) of PNIPAM are favorable to the adsorption of phosphate ions by PNIPAM/SiO_2_ hybrid particles, and the maximum adsorption capacity can reach 287 mg/g (at pH = 5 and 15 °C). The phosphate imprinted polymer/inorganic hybrid material is expected to be put to use in the fields of phosphate ions adsorption, separation, and recovery.

## 1. Introduction

Washing products, dairy processing, and the use of agricultural fertilizer typically produce the discharge of phosphate ions, which result in an increase in the content of phosphate in the water environment. The excessive discharge of phosphorus leads to the eutrophication and deterioration of water quality, giving rise to environmental pollution [1,2,3]. In accordance with the EU Water Framework regulation, phosphorus content in water is not allowed to exceed 0.1 mg/L [4]. Accordingly, the development of functional materials with high selectivity and high capacity to adsorb phosphate anions in water has been a hot issue for researchers.

Inorganic phosphate ion is an oxygen-containing anion, playing an important role in biological, environmental, and chemical processes. In comparison with cations, a phosphate anion has a larger radius, a four-sided geometric configuration, and a strong tendency of solvation. There exists a balance among H_3_PO_4_, H_2_PO_4_^−^, HPO_4_^2−^, and PO_4_^3−^ (p*K*a is 2.1, 7.2, and 12.3) in different pH aqueous solutions, and the hydration capacity and ion radius of the three anions are also different [5]. When pH > 12.3, the form of PO_4_^3−^ mainly exists; when pH is in the range of 7.2–12.3, the form HPO_4_^2−^ is present; and, when pH ranges from 2.1 to 7.2, H_2_PO_4_^−^ mainly exists. When phosphate anions exist in the form of H_2_PO_4_^−^, it is more beneficial for adsorption and sensing research [6]. 

The materials put to use for the adsorption of phosphate anions can be divided into inorganic materials [7,8], organic materials [6,9,10], and organic/inorganic hybrid materials [11]. Kim et al. [7] explored the removal and recycling of phosphate anions from water by layered yttrium hydroxide, followed by finding that this adsorbent material showed advantages of high adsorption capacity, high efficiency, and high reliability. Huang et al. [8] reported a method for the removal of oxygen-containing anions, for instance, phosphate, with the use of sand coated with low cost metal (hydrogen) oxide. The effects of pH value of solution, grain size of sand, coating efficiency, and mineral type on adsorption process were studied and evaluated. Weng et al. [9] studied the interaction of humic acid, and fulvic acid with phosphate anions on goethite surfaces. The results clearly suggest that humic acid and fulvic acid both have different interactions with the phosphate anion. Paltrinieri et al. [11] prepared magnetic Fe_3_O_4_ nanoparticles coated with polyallylamine hydrochloride (PAH) and PAH functionalized with guanidinium groups, in addition to investigating the adsorption performance of polyelectrolyte-modified inorganic particles under alkaline conditions. The results revealed the fact that the magnetic particle material modified with functional polyelectrolytes had the potential to remove phosphate anions, and the adsorption capacity of the modified magnetic particles at pH 5, 8 and 10 was 4 mg/g, 3.6 mg/g, and 3.7 mg/g, correspondingly. In view of the importance of adsorption, separation, and recycling of phosphate anions, it is quite essential to develop and prepare materials with high phosphate adsorption capacity. The design of molecular structures that can be selectively combine with phosphate anions, constitutes the key to the preparation of phosphate adsorbent materials.

In recent years, molecular imprinting technology has attracted the attention of researchers [12,13,14,15,16]. Its characteristics of structural designability, specific recognition performance, and wide applicability are applied to the fields of separation [17,18], sensing [19,20,21,22], and catalysis [23]. Dickert et al. [24] employed a sol-gel method for the preparation of molecular imprinting films, which was put to use as a sensing coating of quartz crystal microbalance to detect K^+^ in water. Hart et al. [25] made use of acrylamide as a functional monomer in order to prepare molecularly imprinted polymers in the water-rich phase for the separation of histidine-based polypeptides, along with a comparison of the effect of different metal ions, which included Ni^2+^, Cu^2+^ and Zn^2+,^ on imprinting effects. The experimental results shed light on the fact that Ni^2+^ had the strongest specific recognition effect on polypeptide molecules. Iskierko et al. [26] prepared conductive molecularly imprinted polymer films with the high surface area by means of sacrificial metal-organic frameworks, and functionalized and cross-linked monomers, which were put to use for the high-sensitive detections of lipid transport proteins.

Among numbers of molecularly imprinted materials, the most promising ones for phosphate adsorption are molecularly imprinted micro/nano particles. The molecularly imprinted particles have the advantages of large specific surface area, easy modification, and so on. For instance, Poma et al. [27] prepared deoxyadenosine imprinted polymer particles, showing the ability to recognize deoxyadenosine; Zhao et al. [28] successfully prepared molecularly imprinted polymer composite microspheres on the basis of quantum dots by means of a simple and multifunctional ultrasonic assisted packaging method, which is different from that of molecularly imprinted polymers based on hydrogen bonds. The composite microspheres, dependent on the interaction of van der Waals and hydrophobic forces, exhibit an excellent selectivity for pesticide detection in aqueous media. In particular, porous materials have the advantages of large specific surface area, uniform pore size distribution, and ordered pore structure. For instance, Chen et al. [29] prepared porous silica particles by means of phosphate imprinting. The molecularly imprinted mesoporous materials exhibit the advantages of excellent selectivity, tolerance to interference, fast binding equilibrium, high binding ability, etc. It makes molecularly imprinted mesoporous materials an ideal adsorbent for the selective enrichment of phosphopeptide. Silica surfaces exposed to water solutions are known for obtaining a negative surface charge density due to the dissociation of silanol (Si–OH) groups [30]. Surfaces with a negative charge results in electrostatic repulsion with the same negative phosphate anions, impacting the adsorption process of anions on the surface of silica. Chibowski et al. [31] discovered the fact that the adsorption of choline phosphate onto the surface of silica microspheres by electrostatic and hydrogen bonding is capable of changing the charge properties and average particle size of silica surface. Shi et al. [32] grafted poly (ethylene glycol) copolymer containing positively charged quaternary ammonium on the surface of mesoporous silica nanoparticles by the living free-radical polymerization and “grafting from” method. The results indicate that the diameter of these mesoporous micron particles in solution decreases greatly, and the Zeta potential increases significantly. Accordingly, it is quite urgent and important to modify the surface of silica microspheres in order to make them more favorable for the adsorption of phosphate anions.

Inorganic silica microspheres are generally modified by physical adsorption [33,34,35] or chemical grafting method [36,37,38]. This surface modified silica is put to extensive use in the field of catalysis, adsorption, and separation [39,40,41,42,43]. Despite the fact that the physical method is simple, it is easy to lead to fall off of the polymer chains from the silica surface under the complicated condition, for instance, pH change. In the chemical method, polymers can be grown from initiators anchored onto the surface, and core-shell structures with a controllable polymer layer can be chemically formed [44]. As compared with the physical method, the chemical grafting method provides core-shell structures that are more effective to change the charge and performance of the silica surface, besides having advantages of higher stability and being easier to control.

It is well known that the PNIPAM polymer and its derivative hydrogels have temperature-sensitive properties, suggesting that there is a low critical solution temperature near 32 °C [45]; the introduction of thermosensitive polymers onto the surface of molecularly imprinted mesoporous silica particles has the potential to improve the surface properties of inorganic particles. In the meantime, the temperature-sensitive polymers can achieve different adsorption properties of phosphate ions at different temperatures, which is similar to the temperature sensitive switch. The natural negative charges existing on the silica surface lower phosphate ion adsorption. The modification of the silica surface with positive charged ligands appears to be a simpler solution as compared with a thermosensitive polymer. The advantage for PNIPAM-based hydrogel is its thermo-sensitivity, which can make the adsorption process stimuli-responsive under temperature control. In comparison with the positively charged ligand, the PNIPAM layer is capable of acting as a temperature-controlled channel for phosphate anion transporting. A study of the preparation and properties of such intelligent materials have been an important research topic. Cheng et al. [46] prepared the Poly(*N*-isopropylacrylamide-*co*-acrylamide-*co*-maleic acid) (P(NIPAM-AM-MA)) hydrogel by free radical polymerization, accordingly discovering that the adsorption behavior of copper(II) (Cu^2+^) ions on P(NIPAM-AM-MA) hydrogel is temperature- and pH-dependent. This pH and temperature sensitive hydrogel is likely to be utilized for the water purification and enrichment of heavy metal ions. This research work aimed at combining the advantages of thermosensitive polymer materials and molecular phosphate imprinted mesoporous silica particles, together with developing the phosphate imprinted polymer/inorganic hybrid material, which is expected to be useful in the fields of phosphate ions adsorption, separation, and recovery.

For the purpose of improving the surface charge property and adsorption efficiency of silica particles, we make use of TMSPMA to modify the phosphate imprinted SiO_2_ particles and introduce a double bond. With the use of NIPAM as the main monomer and K_2_S_2_O_8_ as the initiator, the temperature sensitive PNIPAM/SiO_2_ of hybrid structures were prepared. Fourier transform infrared spectroscopy (FT-IR), thermogravimetric analysis (TGA), nitrogen adsorption-desorption test, laser particle size analysis, and transmission electron microscopy (TEM) were employed to characterize mesoporous SiO_2_ particles and PNIPAM/SiO_2_ hybrid structured particles. The effects of phosphate concentration, pH value, and adsorption temperature on adsorption properties of phosphate anions in water by PNIPAM/SiO_2_ hybrid structured particles were investigated with the help of a UV-visible spectrophotometer.

## 2. Materials and Methods

### 2.1. Materials

*N*-isopropyl acrylamide (NIPAM, 99%), 3-(methoxymethylsilyl) propyl methacrylate (TMSPMA 98%), and potassium persulfate (KPS 99%) were bought from J&K Chemical Ltd. (Beijing, China) and used as received; analytical grade tetraethoxysilane (TEOS, ≥28%, SiO_2_) was bought from Shanghai Lingfeng Chemical Reagent (Shanghai China); 3-ureidopropyltriethoxysilane (UPTES, 40.0–50.0%, methanol solution) was obtained from Aladdin Reagent (Shanghai, China). *N*-Cetyltrimethylammonium bromide (CTAB, 99%) was purchased from Shanghai Richjoint Chemical Reagents (Shanghai, China). Sodium phosphate tribasic dodecahydrate, potassium dihydrogen phosphate, methanol, sodium hydroxide (NaOH), ascorbic acid, antimony potassium tartrate, and hydrochloric acid (HCl) were of analytical grade and were purchased from Sinopharm Chemical Reagent (Beijing, China); Ammonium molybdate tetrahydrate (99%) was purchased from Shanghai Shenbo Chemical (Shanghai, China). Deionized water, which was purified by a Millipore Milli-Q system, was put to use in all experiments.

### 2.2. Preparation of Phosphate-Imprinted Mesoporous Silica Microsphere

0.3 g of CTAB and 0.084 g of NaOH were dissolved into 144 mL of H_2_O in a 500 mL flask. The solution was heated to 80 °C in an oil bath by a magnetic agitator. Following a period of 15 min, 0.76 g of Na_3_PO_4_·12H_2_O was added first, then 1.07 mL of TEOS, 467 μL UPTES, and 100 μL ethanol were fed one by one. A white solution was obtained after a 24 h reaction and was filtered by circulating the water vacuum pump. The white powder was collected and dried at 60 °C in vacuum oven. The templates, which included CTAB and Phosphate anions, were removed from the dried powder by Soxhlet extraction. The extraction solvent was a mixture of 1.5 mL of HCl and 150 mL of methanol. Subsequent to the Soxhlet extraction for 24 h, the powder was dried in a vacuum oven at 60 °C to obtain phosphate imprinted mesoporous silica microspheres.

### 2.3. Preparation of PNIPAM/SiO_2_ Hybrid Particles

0.1 g of phosphate-imprinted mesoporous silica microspheres were placed in a 150-mL flask; subsequent to that, 45 mL of H_2_O and 200 mL of TMSPMA were added in order to form a mixture, which was allowed to blow N_2_ and react at 25 °C for 24 h. The modified silica microspheres were dispersed into 30 mL of water and the dispersion was obtained by the ultrasonic oscillation for 1 h. Following the dispersion heated to 70 °C in an oil bath, 0.02 g of K_2_S_2_O_8_ was added. After blowing N_2_ for 15 min, 0.1 g of NIPAM was added and the reaction was allowed to carry out for 12 h. Eventually, the milky solution was obtained and the unreacted monomers and other water-soluble molecules were removed by the dialysis (dialysis tubing, *M*_w_ cut off 14,000) against the deionized water for 2 days. After every 12 h, the water outside the tubing was replaced. The final dispersion of phosphate-imprinted PNIPAM/SiO_2_ hybrid particles was obtained for further measurements.

### 2.4. Phosphate Adsorption Experiment

Firstly, a certain concentration (0.05, 0.02, and 0.03 mg/mL) of phosphate solution was prepared in a 250 mL beaker. The solution of HCl or NaOH (concentration 1 M) was put to use for adjustment to the desired pH value. The beaker was placed on a magnetic agitator and the temperature was set for the adsorption experiments. PNIPAM/SiO_2_ hybrid particles contained in the dialysis tubing membrane were allowed to adsorb phosphate anions from the solution for two days. Following the adsorption, the phosphate concentration of the solution in the beaker before and after adsorption was determined with the help of the phosphor-molybdenum blue method (see Figures S1 and S2 in Supplementary Materials) with the standard reference curve (Y = 194.28571X + 0.00479 (R^2^ = 0.97807)).

The calculation method of the phosphate binding capacity of PNIPAM/SiO_2_ hybrid particles is presented as hereunder:

(1) $$Q_{e}=\frac{C_{0}V_{0}-C_{1}V_{1}}{m}$$

wherein, *Q_e_* indicates the phosphate binding capacity of PNIPAM/SiO_2_ hybrid particles, and the unit mg/g; *C*_0_ represents the concentration of phosphate solution in the beaker before the adsorption, and *C*_1_ denotes the concentration of phosphate solution in the beaker after the adsorption (mg/mL). *V*_0_ suggests the initial volume of the phosphate solution in the beaker (mL). *V*_1_ indicates the sum of the volume of the final solution in the beaker and the volume of the particle dispersion in dialysis tubing membrane (mL). *m* is the mass of the PNIPAM/SiO_2_ hybrid particles (g). Note that all the measurement was repeated for three parallel experiments, and the averaged value was utilized as the final binding capacity.

#### 2.4.1. The Adsorption of PNIPAM@SiO_2_ with Phosphate Anions over Time

The phosphate solution with a concentration of 0.18 g/mL was prepared and the required pH value was adjusted by HCl or NaOH solution with a concentration 1 M. 10 mL of the PNIPAM/SiO_2_ particle dispersion having a concentration of 5 mg/mL in a dialysis tubing membrane was placed in the phosphate solution for the purpose of adsorbing the phosphate anion, and 1 mL phosphate solution was taken out for measurement every other time. The concentration of phosphate solution was determined by the molybdenum blue method mentioned earlier.

When the phosphate adsorption of the PNIPAM/SiO_2_ hybrid particles increases with time, the pseudo-second-order kinetic equation [11,47] can be utilized for a nice fitting. This is owing to the fact that this equation is especially suitable for phosphate solutions with lower initial concentration.

(2) $$\frac{dQ_{t}}{dt}=k_{2}{\left(Q_{e}-Q_{t}\right)}^{2}$$

(3) $$\frac{t}{Q_{t}}=\frac{1}{k_{2}Q_{e}^{2}}+\frac{1}{Q_{e}}t$$

wherein, *t* indicates the adsorption time, *Q_t_* denotes the phosphate adsorption amount at time *t*, *Q_e_* suggests the phosphate adsorption capacity at equilibrium, and *k*_2_ suggests the pseudo-second-order rate constant.

#### 2.4.2. The Phosphate Adsorption of PNIPAM/SiO_2_ Particles Varying with the Initial Phosphate Concentration

The phosphate solutions, with three different concentrations (0.05, 0.02, and 0.03 mg/mL), were prepared in a 250-mL beaker, correspondingly. The HCl or NaOH solution with a concentration of 1 M was put to use for the adjustment of the required pH value. Moreover, an amount of 10 mL of the PNIPAM/SiO_2_ particle dispersion with a concentration of 5 mg/mL in a dialysis tubing membrane was placed in the phosphate solution in order to adsorb the phosphate anion, and the effect of the initial phosphate concentration on adsorption was investigated.

#### 2.4.3. The Effect of Solution pH on the Phosphate Adsorption of PNIPAM/SiO_2_ Particles

Three parts of the phosphate solution with a concentration of 0.02 mg/mL were prepared in 250 mL beaker correspondingly, and their pH (5, 7, and 10) were adjusted by using HCl or NaOH (concentration 1 M). The three groups of PNIPAM/SiO_2_ particle dispersion with a concentration of 5 mg/mL were placed into the solution for adsorbing phosphate anions for 30 h, and the effect of pH on adsorption was investigated.

### 2.5. Characterizations

#### 2.5.1. FT-IR Spectroscopy

The samples were dried into powder, mixed with KBr powder, fully ground and pressed into transparent sheets for the purpose of testing. The infrared spectra of the samples were measured with the use of the Fourier transform infrared spectrometer (Avatar 370, Thermo Nicolet Corporation, Madison, WI, USA). The scanning range is between 4000 and 500 cm^−1^ with a resolution of 4 cm^−1^.

#### 2.5.2. Thermogravimetric Analysis

The TG209F1 thermogravimetric analyser of NETZSCH Group (Selb, Bavaria, Germany) was employed and the sample was scaled with a mas approximately from 3 to 6 mg. The heating rate is 20 °C/min; the flow rate of nitrogen atmosphere is 50 mL/min; and the scanning temperature ranges from 30 to 600 °C.

#### 2.5.3. Specific Surface Area and Pore Size Measurements

Nitrogen adsorption−desorption measurements of the sample were carried out on a TriStar 3020 type adsorption instrument produced by Micromeritics Instrument Corporation, Norcross, GA, USA. The Brunauer−Emmett−Teller (BET) method was adopted for the calculation of the surface areas, and the Barrett−Joyner−Halenda (BJH) method was employed for the determination of pore size distributions.

#### 2.5.4. Particle Size Test

The sample solution was diluted prior to the measurements. Normally, the silica particle solutions in water should be ultrasonicated for 30 min prior to the laser particle size analysis of the silica samples. A laser particle analyser (Nano-ZS, Malvern Panalytical, Malvern, UK) was employed to observe the particle size and zeta potential of the samples at 25 °C and at a scattering angle of 90°.

#### 2.5.5. Electron Spectroscopy

The morphology and size of the sample were observed by scanning electron microscope (SEM) of JSM-6510 model of JEOL (JEOL Ltd., Tokyo, Japan); in addition, the liquid droplets to be tested were added to a 1 cm × 1 cm glass. After a few minutes, the excess solution was gently sucked out with filter paper and the sample was dried in the air. The morphology and dispersion of SiO_2_ microspheres before and after the modification were obtained by means of scanning electron microscope. Prior to the SEM observation, the sample was coated with gold.

The structure and morphology of the samples were also observed by field emission transmission electron microscope (JEM2100F, JEOL, Japan), and the working voltage was accelerated by 200 KV. The mesoporous silica and the phosphate imprinted PNIPAM/SiO_2_ hybrid particles were dispersed in ethanol, followed by dropping on the copper network surface, which contained ultra-thin carbon film as a support frame, after the ultrasonic dispersion. The surface morphology and size were tested following the natural drying.

#### 2.5.6. The Phosphate Adsorption Performance of PNIPAM/SiO_2_ Hybrid Particles

Ultraviolet and visible (UV-vis) spectrophotometer (MV1800, Shanghai Yanrun Optical Machine Technology Co., Ltd. Shanghai, China) was employed for the detection of the absorbance of the solution. In this experiment, phosphate anions were blue-colored with the help of the phosphomolybdate blue method, and the phosphate concentration before and after the adsorption [48] was shown by the change of absorbance.

## 3. Results and Discussion

### 3.1. Preparation and Characterization of Phosphate-Imprinted SiO_2_ and PNIPAM/SiO_2_ Particles

The phosphate imprinted silica microspheres were prepared by the three steps presented as hereunder, which included the first formation of the complex between the template phosphate and the rod-like CTAB micelle (mesoporous template) by the electrostatic interaction, followed by the imprinting with silicon precursor TEOS and UPTES, and finally removing template molecules. By means of the three-step synthetic pathway, the combination of the double templates including phosphate and CTAB rod resulted in mesoporous structures and molecular imprinting cavities on different scales inside the silica microspheres. The imprinting of phosphate anions was printed on the inner wall of the mesoporous structure. This unique configuration is capable of providing more sites, together with a high specific surface area, for the phosphate binding. Phosphate-imprinted mesoporous silica microspheres are able to adsorb phosphate anions [29,49], besides acting as an ideal adsorbent for phosphate separation and recovery. As discussed above, the surface charge property of silica microspheres can greatly impact the binding of phosphate anions with the silica microspheres. Accordingly, the modification of the SiO_2_ microsphere with thermosensitive PNIPAM layers was carried out in the following procedure. Firstly, TMSPMA was put to use as a silane coupling agent in order to introduce double bonds on the surface of SiO_2_ by the chemical bonding. Thereafter, in the presence of NIPAM monomer and KPS as initiator, PNIPAM modified SiO_2_ particles (PNIPAM/SiO_2_) were fabricated by the free radical polymerization.

**Figure 1:**
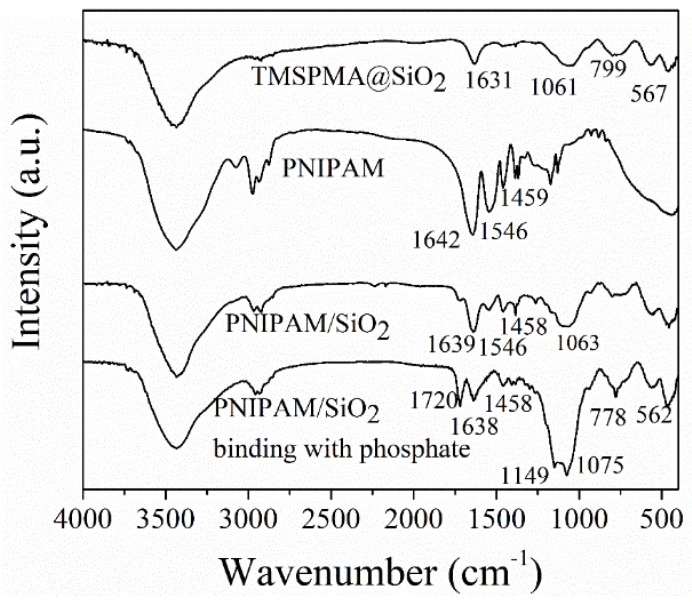
FT-IR spectra of TMSPMA@SiO_2_, PNIPAM microgels, PNIPAM/SiO_2_ hybrid particles, and PNIPAM/SiO_2_ hybrid particles binding with phosphate.

Figure 1 sheds light on the FT-IR spectra of the SiO_2_ microspheres modified with TMSPMA, PNIPAM microgels, phosphate imprinted PNIPAM/SiO_2_ hybrid particles, and PNIPAM/SiO_2_ hybrid particles binding with phosphate anions. As evident from Figure 1, the spectrum of the modified SiO_2_ (TMSPMA@SiO_2_) showed the characteristic peaks (−C=O) at 1631 cm^−1^, (Si–O) 1061 cm^−1^, and (Si–C) 799 cm^−1^, proving that TMSPMA has successfully grafted to the surface of SiO_2_ microspheres. In accordance with the spectrum of PNIPAM, the characteristic absorption peaks at 1459, 1546, and 1642 cm^−1^ correspond to the C=O stretching vibration, N–H stretching vibration, and methyl asymmetric bending vibration. As suggested by the spectrum of PNIPAM/SiO_2_, the characteristic peaks belonging to NIPAM units and TMSPMA modified SiO_2_ are still present, confirming the fabrication of PNIPAM/SiO_2_ hybrid particles. In comparison with PNIPAM/SiO_2_ hybrid particles, the PNIPAM/SiO_2_ hybrid particles binding with phosphate manifested the absorption peak at 1149 cm^−1^, belonging to the asymmetric stretching vibration of phosphate. Also, the absorption peak at 778 cm^−1^ was assigned to the in-plane bending vibration of phosphate, and the one at 1720 cm^−1^ corresponded to the P–O stretching vibration in the spectrum of PNIPAM/SiO_2_ hybrid particles binding with phosphate. That is why FT-IR spectra confirmed the successful synthesis of PNIPAM/SiO_2_ hybrid particles, and their ability to bind with phosphate anions.

**Figure 2:**
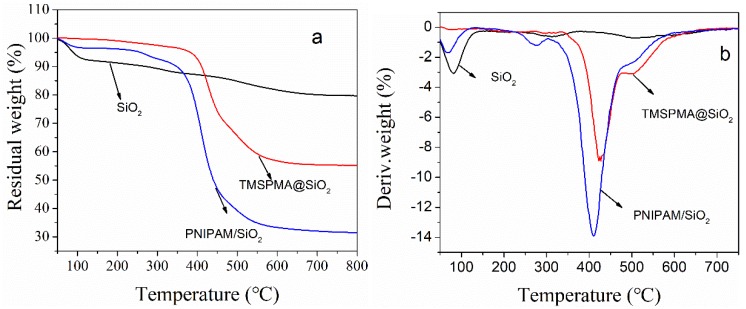
TGA (**a**) and DTG (**b**) thermograms of phosphate imprinted SiO_2_, TMSPMA@SiO_2_ and PNIPAM/SiO_2_ hybrid particles.

With the help of TGA tests of polymer/inorganic composite particles, it can be confirmed whether the PNIPAM chains were grafted on the phosphate imprinted mesoporous silica microspheres. Subjected to high temperature conditions, organic PNIPAM chains on SiO_2_ microspheres were decomposed, and the remaining high-temperature resistant materials were the inorganic silica. Figure 2 shows the TGA and DTG thermograms of phosphate imprinted SiO_2_, TMSPMA@SiO_2_ and PNIPAM/SiO_2_ hybrid particles. As evident from Figure 2, the residual mass fraction of phosphate imprinted mesoporous SiO_2_ is 81%, and the mass loss of 21% is associated with the mass of trace organic matter and water that are not resistant to the high temperature. The residual mass content of TMSPMA@SiO_2_ is 55.2%, and the weight loss of 45% belongs to the unstable TMSPMA and water molecules. The residual mass of PNIPAM/SiO_2_ hybrid particles was 31.41%, and the mass loss of 69% was assigned to the unstable polymers and water. Both the NIPAM monomer and the PNIPAM polymer are materials that are not resistant to the high temperature, and there will be losses at the high temperature. The remaining materials are high-temperature resistant silica. Accordingly, it is proved that SiO_2_ and PNIPAM chains are successfully bound to each other. Figure 2b shows DTG curves that correspond to the three materials observed in Figure 2a. Apparently, the decomposition rate of the three substances changes with the temperature. The mesoporous silica SiO_2_ manifests the weight loss before 100 °C, which is due to water evaporation. This temperature is lower than the degradation peak (T_max_) values that include 400 °C for TMSPMA@SiO_2_ and 420 °C for PNIPAM/SiO_2_ hybrid particles. Therefore, it can be concluded that PNIPAM/SiO_2_ hybrid particles do have inorganic silica and organic polymer components. As evident from Figure 2, the grafted TMSPMA takes up ~24% of the PNIPAM/SiO_2_ weight, while the grafted PNIPAM chains take up another ~25% of the PNIPAM/SiO_2_ weight. It suggests that the degree of polymerization for the grafted PNIPAM is probably low in the PNIPAM/SiO_2_ particles in our work.

**Figure 3:**
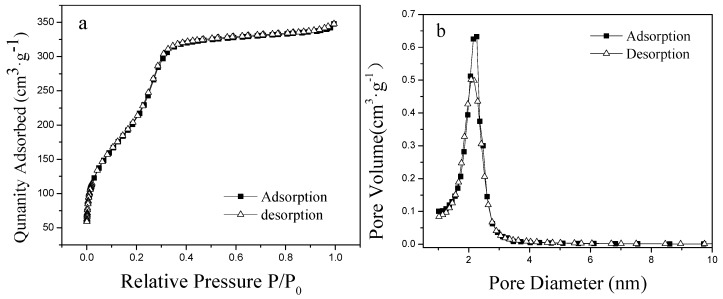
N_2_ adsorption-desorption isotherms (**a**) and pore size distribution (**b**) of the phosphate imprinted mesoporous SiO_2_ microspheres.

Figure 3a sheds light on the nitrogen adsorption-desorption isotherm of phosphate-imprinted mesoporous SiO_2_ microspheres. As suggested by Figure 3a, the nitrogen adsorption isotherm of silica increases sharply when the relative pressure is <0.4. This constitutes the adsorption of micropores, and the increase in the adsorption between 0.4 and 1.0 can be regarded as the adsorption of large micropores, transition pores, and middle pores. At a relative pressure >0.4, the adsorption curve increased slowly, which is an indication of the characteristics of microspore as the main material, meanwhile having more medium pores. Figure 3b shows the pore diameter distribution of phosphate-imprinted SiO_2_ microspheres. For the average mesopore diameter, calculated by BJH is 2.3 nm, the pore volume is 0.5–0.65 cm^3^/g, and the BET specific area is 883 m^2^/g. This is in line with the typical characteristics of mesoporous materials.

**Figure 4:**
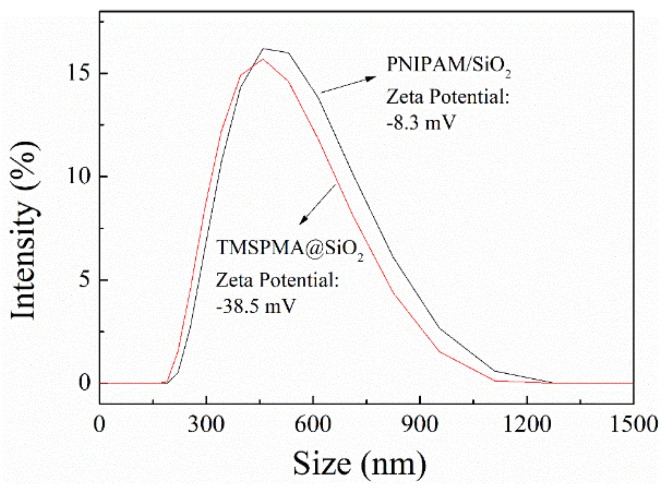
Particle size distribution curves of TMSPMA@SiO_2_ and PNIPAM/SiO_2_ particles (pH = 7, T = 25 °C).

Figure 4 sheds light on the particle size distribution curves of TMSPMA@SiO_2_ and PNIPAM/SiO_2_ hybrid particles. As evident from Figure 4, the particle size of TMSPMA@SiO_2_ is distributed between 200 and 1100 nm, concentrated at approximately 500 nm, and the average particle size is 459 nm. As highlighted in Figure 4, the particle size of PNIPAM/SiO_2_ hybrid particles is between 200 nm and 1100 nm, concentrated at approximately 500 nm, and the average particle size is 484 nm. In accordance with the results of particle size distribution, the modified phosphate-imprinted TMSPMA@SiO_2_ and PNIPAM/SiO_2_ hybrid particles were both particles at the micron meter scale.

Laser particle size analyser data revealed the fact that TMSPMA@SiO_2_ microsphere has a zeta potential of −38.5 mV, due to the dissociation of silanol (Si–OH) groups, which results in negatively charged surfaces. Nevertheless, the PNIPAM/SiO_2_ hybrid particle has a zeta potential of −8.3 mV, which is higher as compared with that of the TMSPMA@SiO_2_ microsphere. This is owing to the fact that most of the Si–OH groups on the silica surface are involved in the grafting reaction, and the number of Si–OH groups is reduced, while the PNIPAM chains are grown from the sites by means of free radical polymerization. However, since surface grafting polymerization makes use of the KPS initiator, the existence of the initiator KPS residue also gives the PNIPAM/SiO_2_ hybrid particle negative charges. A conclusion can be reached that, by means of the surface chemical modification, the charge property and particle size of phosphate imprinted mesoporous SiO_2_ microspheres can be changed, which benefits the next phosphate adsorption experiments.

**Figure 5:**
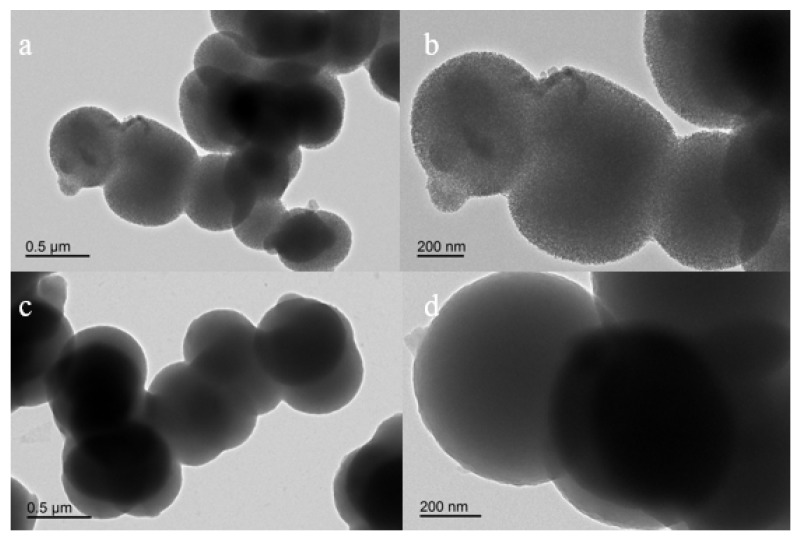
TEM images of phosphate imprinted SiO_2_ (scale bar: (**a**) 500 nm, (**b**) 200 nm) and PNIPAM/SiO_2_ hybrid particles (scale bar: (**c**) 500 nm, (**d**) 200 nm).

Figure 5 presents TEM images of phosphate imprinted SiO_2_ and PNIPAM/SiO_2_ hybrid particles. As evident from Figure 5a,b, the particle size of phosphate imprinted SiO_2_ is approximately 500 nm. The mesoporous SiO_2_ microspheres are composed of several smaller nanoparticles. Each SiO_2_ microsphere contains a rough surface, together with a mesoporous structure inside, which is in line with the results of the N_2_ adsorption-desorption isotherm. Figure 5c,d shed light on TEM images of PNIPAM/SiO_2_ hybrid particles, of which particle size is approximately 500 nm and the surface is comparatively smoother [50]. It is impossible to observe that the big PNIPAM/SiO_2_ hybrid particle is composed of smaller particles. It can be also observed that the color of the center of the PNIPAM/SiO_2_ particle is much darker in comparison with the edge of the same sphere, which is due to the higher electron density in the center. In comparison with silica, the PNIPAM/SiO_2_ particles manifested a denser structure, suggesting the grafting of the polymers onto the silica structures. If the polymers have not covered the silica particles, the same porous structures as silica could be observed in the image of PNIPAM/SiO_2_ particles. Figure S3 in Supplementary Materials presents the SEM images of silica spheres before and after impregnation with PNIPAM. Changes in the shape and size of the structures actually confirm the grafting of PNIPAM layers onto the silica surface, based on two points: Firstly, a particle agglomerate was observed, which could be attributed to the high concentration of the particles and surface tension effect during the drying procedure for SEM measurement. Secondly, polymers were chemically attached onto the silica surfaces owing to free radical polymerization. The water-soluble PNIPAM polymers hardly remained after the separation and purification, such as filtration and dialysis against water. Figures S4 and S5 in Supplementary Materials illustrate TEM images of PNIPAM/SiO_2_ hybrid particles and the size distribution of the phosphate-imprinted SiO_2_ microspheres. It implies that the final product is absolutely not a mixture of silica particles and polymer excess. The FT-IR and TGA results highlighted earlier confirmed the chemically grafting of PNIPAM layer onto the SiO_2_ microspheres.

### 3.2. Adsorption of Phosphate Anions in Water by Phosphate Imprinted SiO_2_ and PNIPAM/SiO_2_ Hybrid Particles

The phosphate-imprinted mesoporous SiO_2_, PNIPAM microgel and PNIPAM/SiO_2_ hybrid particle (the mass concentration of dispersible solution is 5 mg/mL) were employed for adsorbing phosphate anions in the solution with an initial phosphate concentration of 0.2 mg/mL, and the adsorption time was 30 h. The adsorption time was set long enough, aimed at ensuring that the adsorption equilibrium is achieved. Subsequent to that, the changes in the phosphate concentration of the aqueous solution, before and after the adsorption, were measured by the phosphomolybdenum blue method. As evident from Table 1, adsorption amounts of phosphate-imprinted mesoporous SiO_2_, PNIPAM microgel, and PNIPAM/SiO_2_ hybrid particle in water were 20.0 ± 1.2 mg/g, 55.0 ± 2.7 mg/g, and 271.0 ± 13.5 mg/g (as calculated by PO_4_^3−^), correspondingly. There are numerous imprinting cavities in the phosphate-imprinted mesoporous SiO_2_, which can match the spatial configuration of phosphate anions, besides having multiple interaction points. However, the adsorption amount of phosphate-imprinted mesoporous SiO_2_ is only 20 mg/g due to the electrostatic repulsion between the negatively charged silica surface and the same charged phosphate anions. Consequently, it is difficult for the phosphate anions to enter the mesoporous silica microsphere, leading to a comparatively smaller amount of adsorption for mesoporous SiO_2_. PNIPAM microgels are a kind of three-dimensional cross-linked network structure with size that ranges from several nanometers to micrometers, together with excellent hydrophilic and swelling-deswelling properties. The preparation and characterization of PNIPAM microgels used here can be found in Figure S6 in Supplementary Materials [51]. The adsorption of phosphate anions by PNIPAM microgels is primarily driven by hydrophilic interaction and hydrogen bonding. The positive performance of PNIPAM microgels is not quite strong, and does not show any specific adsorption for phosphate anions. However, the whole PNIPAM microgels can act as a hydrophilic container with a cross-linking network of a high swelling degree and high specific surface area, which is beneficial for the acquirement of the phosphate anions. In addition, the phosphate anions have weak affinities for amide moieties of PNIPAM chains, which are hydrogen bond donor groups [52]. Both factors contributed to the phosphate adsorption ability of 55 mg/g for PNIPAM microgels. The phosphate adsorption capacity of PNIPAM/SiO_2_ hybrid particles has the potential to reach 271 mg/g, which is 13 times the value of phosphate-imprinted mesoporous SiO_2_. It is worth observing that the adsorption capacity of PNIPAM/solid SiO_2_ to phosphate ion is 61 mg g^−1^, which is way lower than the value of 271 mg g^−1^ of the PNIPAM/imprinted SiO_2_ particles (see Supplementary Materials, Figure S7). This also suggests a significant effect of phosphate-imprinted silica structures in this work. Paltrinieri et al. [11] modified the magnetic Fe_3_O_4_ nanoparticles with polyelectrolyte (PAH-Gu) containing guanidinium groups, in addition to investigating the phosphate adsorption performance of the particles in water. They discovered that the new PAH-Gu@ Fe_3_O_4_ material can effectively absorb phosphate anions. At pH = 8, the adsorption capacity can reach 3.6 mg/g (in terms of P), translating to a phosphate concentration of 11.03 mg/g. The adsorption capacity of PNIPAM/SiO_2_ hybrid particle in our work is 24 times greater as compared to that of PAH-Gu@Fe_3_O_4_ material, which is associated with the role of PNIPAM structure and molecular imprinting of the internal SiO_2_ structure. The PNIPAM layer on the surface of SiO_2_ microspheres is in a highly swelling state with water, and phosphate anions go through the first layer of the soft and hydrated PNIPAM polymer layer, together with being likely to bind with amide moieties of PNIPAM due to hydrogen bonding and hydrophilic interactions. It is hypothesized that the PNIPAM layer could act as a channel for phosphate anion transporting without directly exposing phosphate anions to the silica surface. Subsequent to that, phosphate anions could enter into the internal mesoporous SiO_2_ microspheres and bind with the imprinting sites. Accordingly, as compared with the phosphate imprinted mesoporous SiO_2_ and PNIPAM microgel, the PNIPAM/SiO_2_ hybrid particle has an excellent phosphate adsorption capacity.

**Figure 6:**
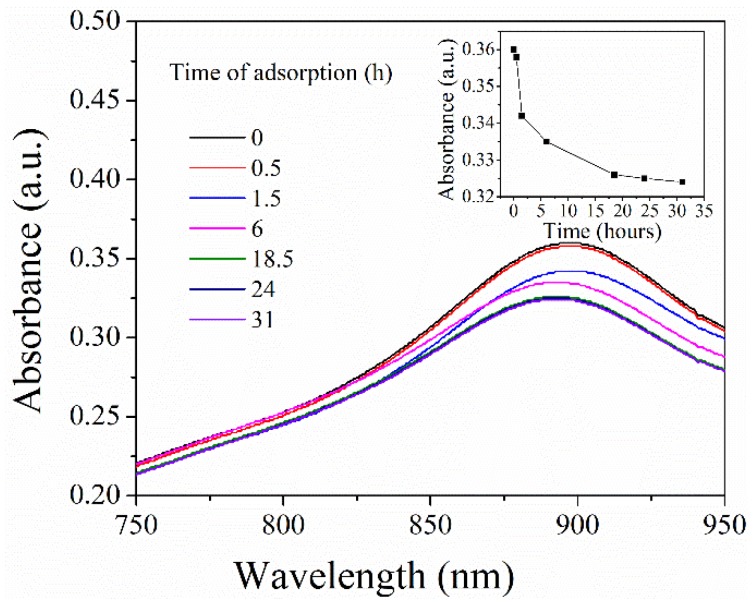
UV-vis absorption spectra of phosphate solutions after adsorption over time (the inset showed the corresponding absorbance at 890 nm as a function of adsorption time).

Figure 6 sheds light on UV-vis absorption spectra of phosphate solutions after adsorption over time (the inset reveals the corresponding absorbance at 890 nm as a function of adsorption time). Notably, PNIPAM/SiO_2_ hybrid particles with a concentration of 5 mg/mL was put to use to adsorb phosphate anions (the initial phosphate concentration of 0.18 mg/mL). As evident from Figure 6, with the increase in adsorption time, there was a decline in the UV-vis absorption intensity of phosphate solution. When the adsorption time reached approximately 30 h, the absorption spectrum was basically overlapped with the previous one at 24 h, which suggested that the concentration of phosphate solution does not change at this time. At this point, the phosphate adsorption of PNIPAM/SiO_2_ hybrid particles reached adsorption saturation.

**Figure 7:**
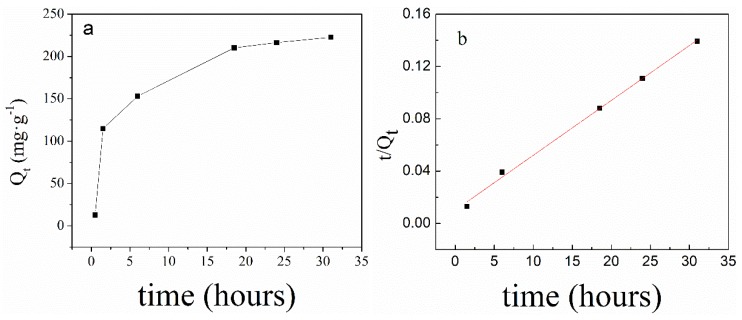
The amount of phosphate adsorbed by PNIPAM/SiO_2_ hybrid particles in aqueous solution over time (**a**) and the adsorption kinetics of PNIPAM/SiO_2_ (**b**) (pH = 7, 15 °C).

Figure 7 illustrated the amount of phosphate adsorbed by PNIPAM/SiO_2_ hybrid particles in aqueous solution over time (a) and the adsorption kinetics of PNIPAM/SiO_2_ (b) at pH = 7 and 15 °C. It requires observation that the PNIPAM/SiO_2_ dispersion, with a concentration of 5 mg/mL and initial phosphate solution of 0.18 mg/mL, was utilized. As evident from Figure 7a, the adsorption of PNIPAM/SiO_2_ hybrid particles to phosphate anions indicates a slow adsorption behavior, which reaches adsorption equilibrium following almost 30 h. In accordance with the curve, when the adsorption reaches equilibrium, the amount of phosphate adsorbed by PNIPAM/SiO_2_ hybrid particles at pH = 7 is 222 mg/g. The pseudo-second-order kinetic Equation (2) can be employed to describe the changes in the amount of phosphate adsorbed as a function of time, which is termed as the best model at a low concentration solution. The linear form of Equation (2) can be described by Equation (3). Figure 7b indicates the adsorption kinetics curve of PNIPAM/SiO_2_ particles. As indicated by 7b, the ratio of t/Q_t_ increases linearly with the increase in time t, which is in line with the curve of the amount of phosphate adsorbed over time, presented in Figure 7a. Subsequent to fitting, the kinetic parameter *K*_2_ is 0.00173 g/mg/h, the adsorption capacity *Q_e_* is 238 mg/g at equilibrium, and the coefficient of determination R^2^ is 0.99656, which suggest quite a high fitting quality.

**Figure 8:**
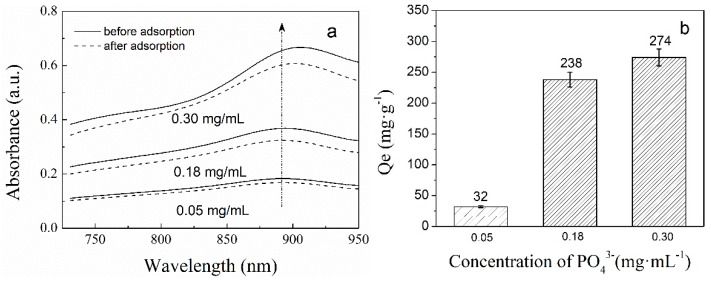
The UV-vis absorption spectra of phosphate solutions before and after adsorption at different initial concentrations of phosphate solutions (**a**) and the final adsorption capacity with various initial phosphate concentrations (**b**).

The effect of the initial concentration of phosphate solutions (0.05, 0.18, 0.3 mg/mL) on the adsorption was investigated in this experiment. Figure 8 presented the UV-vis absorption spectra of phosphate solutions before and after adsorption at different initial concentrations of phosphate solutions (a) and the final adsorption capacity with various initial phosphate concentrations (b). As is evident from Figure 8a, the UV-vis absorption intensity of phosphate solutions, after the adsorption, decreased under the different initial concentrations of phosphate solutions, which suggests the binding of phosphate anions with PNIPAM/SiO_2_. As evident from Figure 8b, when the initial concentrations of phosphate solutions are 0.05, 0.18, and 0.30 mg/mL, the corresponding adsorption capacities of PNIPAM/SiO_2_ particles are 32, 238, and 274 mg/g. It can be concluded that the higher the initial concentration of phosphate solution, the more the amount of phosphate PNIPAM/SiO_2_ particles adsorbed.

**Figure 9:**
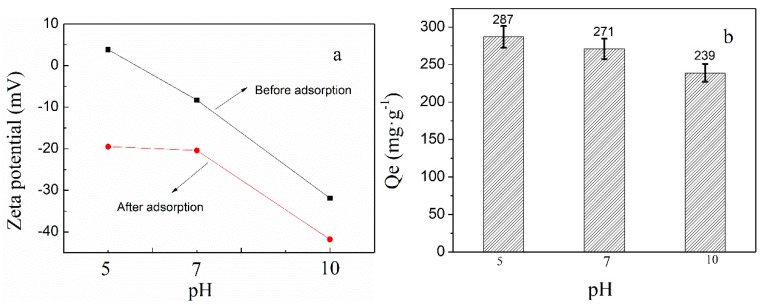
Zeta potential of PNIPAM/SiO_2_ particles before and after adsorption of phosphate anions at different pH (**a**) and phosphate adsorption capacity of PNIPAM/SiO_2_ particles after adsorption at different pH (**b**).

A 10 mL of PNIPAM/SiO_2_ dispersion with a concentration of 5 mg/mL was put to use for the purpose of adsorbing phosphate anions (the concentration of solution was 0.2 mg /mL) for 30 h, and the pH was adjusted to 5, 7 and 10, correspondingly. The effect of pH on the adsorption of phosphate anions by P PNIPAM@SiO_2_ particles was also investigated. Figure 9 demonstrated the Zeta potential of PNIPAM/SiO_2_ particles before and after the adsorption of phosphate anions at different pH (a) and the phosphate adsorption capacity of PNIPAM/SiO_2_ particles at different pH (b). As evident from Figure 9a, Zeta potentials of PNIPAM/SiO_2_ particles before the adsorption of phosphate anions are higher as compared with those obtained after the adsorption at different pH values. As evident from Figure 9b, the amounts of phosphate adsorbed on PNIPAM/SiO_2_ particles at pH 5, 7, and 10 are found to be 287, 271, and 239 mg/g, correspondingly. When pH is 5, the phosphate adsorption amount of PNIPAM/SiO_2_ is observed as the largest. When pH is 5 or the solution is acidic, the presence of H^+^ makes PNIPAM/SiO_2_ particles positively charged, which is confirmed by the Zeta potential value of +3.8 mV as presented in Figure 9a. The role of phosphate imprinting and electrostatic interaction increases the phosphate adsorption amount of PNIPAM/SiO_2_ particles. Following the adsorption of H_2_PO_4_^−^ at pH 5, the Zeta potential of PNIPAM/SiO_2_ particles decreases to −19.5 mV. When pH is 10 or the solution is alkaline, the presence of OH^−^ makes PNIPAM@SiO_2_ negatively charged and its Zeta potential is −31.9 mV. In the meantime, phosphate anions adopt the form of HPO_4_^2−^ with more negative charges, which results in more electrostatic repulsion with PNIPAM/SiO_2_ particles with the same charges and less amount of phosphate adsorbed. At pH 7, the adsorption of phosphate anions with PNIPAM/SiO_2_ particles is somewhere in between.

However, the zeta potential of PNIPAM/SiO_2_ particles at pH = 10 (−31.9 mV) is comparable to that of TMSPMA@SiO_2_ at pH = 7 (−38.5 mV), while the two apparently have drastically different capabilities of absorbing phosphate. It suggests that the enhanced phosphate adsorption of PNIPAM/SiO_2_ particles cannot only be attributed to their less negatively charged surface, as compared with the unfunctionalized and phosphate-imprinted SiO_2_ particles. This is also associated with the role of a PNIPAM shell layer on the imprinted SiO_2_ particles, which is already explained in Table 1.

**Figure 10:**
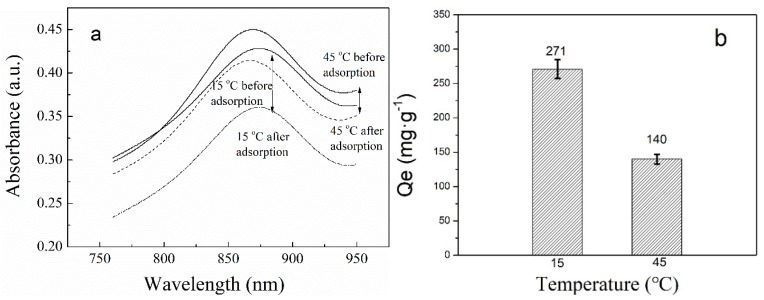
The UV-vis absorption spectra of phosphate solutions before and after adsorption (**a**) and phosphate adsorption capacity of PNIPAM/SiO_2_ particles (**b**) at 15 and 45 °C (pH = 7).

Phosphate anions were adsorbed with the use of PNIPAM/SiO_2_ dispersion in water that had a concentration of 5 mg/mL and phosphate solution having an initial concentration of 0.2 mg/mL. Figure 10 demonstrates the UV-vis adsorption spectra of phosphate solutions before and after the adsorption (a) and the phosphate adsorption capacity of PNIPAM/SiO_2_ particles (b) at 15 and 45 °C. As evident from Figure 10a, the UV-vis absorption intensity of phosphate solutions after the adsorption decreased, and the difference in the absorption intensity before and after the adsorption at 15 °C is much larger in comparison with that obtained at 45 °C. It indicates that the adsorption temperature does impact the phosphate adsorption of PNIPAM/SiO_2_ particles. As evident from Figure 10b, the phosphate adsorption capacities of PNIPAM/SiO_2_ particles at 15 and 45 °C were found to be 271 and 140 mg/g, which can be attributed to the influence of temperature on PNIPAM layers on the SiO_2_ surface. As is known, thermosensitive PNIPAM in the aqueous solution has a low critical solution temperature (LCST) at approximately 31 °C. When the adsorption temperature is lower than the LCST, the PNIPAM shell layer on the SiO_2_ surface swells with the water and PNIPAM chains are elongated, leading to a large hydrodynamic radius of PNIPAM/SiO_2_ particles. It is possible for phosphate anions to pass through the PNIPAM shell layer, together with entering into the binding sites of the SiO_2_ microspheres imprinted. In the meantime, phosphate anions will also be adsorbed into the PNIPAM shell layer owing to the hydrogen bond between phosphate and amide moieties. When the temperature is higher than LCST, PNIPAM layer on SiO_2_ surface are in the collapsed state and PNIPAM chains become more hydrophobic and expels water, which results in a small hydrodynamic radius of PNIPAM/SiO_2_ particles. Accordingly, PNIPAM/SiO_2_ particles in a collapsed state tend to adsorb a small amount of phosphate anions.

## 4. Conclusions

The phosphate imprinted PNIPAM/SiO_2_ particles can be prepared by the first synthesis of imprinted SiO_2_ microspheres and the subsequent “grafting from” polymerization of thermosentive monomer NIPAM. FT-IR spectra, TGA, laser particle size analysis, and TEM confirmed the formation of the PNIPAM/SiO_2_ hybrid structures. BET and BJH tests shed light on the fact that the specific area of phosphate-imprinted SiO_2_ is 883 m^2^/g, and the average pore diameter is 2.3 nm, which indicated the mesoporous structures of SiO_2_ microspheres. With the use of UV-vis spectroscopy, the PNIPAM/SiO_2_ particles manifested an excellent phosphate binding capacity of 271 mg/g at pH 7 and 15 °C, while the phosphate adsorption abilities of imprinted SiO_2_ microspheres and PNIAM microgels are only 20 and 55 mg/g, correspondingly. In addition, the effects of adsorption time, initial concentration of phosphate anions, pH value, and temperature on the phosphate adsorption of PNIPAM/SiO_2_ particles were investigated, and the results revealed the fact that the amount of phosphate adsorbed by PNIPAM/SiO_2_ could reach 287 mg/g at pH 5 and 15 °C. The high adsorption capacity of PNIPAM/SiO_2_ particles is associated with the molecular imprinting of internal SiO_2_ microspheres and structure of the PNIPAM shell layer. PNIPAM shell on the surface of SiO_2_ microspheres is in a highly swelling state with the water, and phosphate anions go through the first layer of soft and hydrated PNIPAM polymer layer, in addition to being likely to bind with amide moieties of PNIPAM owing to hydrogen bonding. It is hypothesized that the PNIPAM layer could act as a channel for phosphate anion transporting without directly exposing phosphate anions to the silica surface. Thereafter, phosphate anions could enter internal mesoporous SiO_2_ microspheres, together with binding with the imprinting sites. Both factors contributed to the high phosphate adsorption ability of PNIPAM/SiO_2_ particles. The polymer/inorganic hybrid imprinting materials are expected to be put to use in the adsorption, separation, and recovery of phosphate anions in water.

## Figures and Tables

**Table 1:** Adsorption of phosphate anions by different adsorbents (pH = 7, 15 °C).

Adsorbent	Imprinted SiO_2_	PNIPAM Microgel	PNIPAM/SiO_2_ Particles
Adsorption capacity (mg/g)	20.0 ± 1.2	55.0 ± 2.7	271.0 ± 13.5

## Supplementary Materials

The following are available online at http://www.mdpi.com/2073-4360/11/2/253/s1, Figure S1: UV-vis spectra of the phosphate standard solutions, Figure S2: A standard curve (Absorbance vs Phosphate concentration), Figure S3: SEM images of phosphate imprinted mesoporous SiO_2_ (Left) and PNIPAM/SiO_2_ (Right), Figure S4: TEM images of PNIPAM/SiO_2_ hybrid particles (a 0.5 μm, b 100 nm), Figure S5: The size distribution of the phosphate imprinted SiO_2_ microspheres. Figure S6: SEM image of PNIPAM microgels, Figure S7: The comparison of phosphate adsorption capacity of phosphate imprinted SiO_2_, PNIPAM microgels, PNIPAM@solid SiO_2_, and PNIPAM@imprinted SiO_2_.

## References

[1] Schroder J., Cordell D., Smit A., Rosemarin A. (2010). Sustainable Use of Phosphorus.

[2] Hudson J.J., Taylor W.D., Schindler D.W. (2000). Phosphate Concentrations in Lakes. Nature.

[3] Conley D.J., Paerl H.W., Howarth R.W., Boesch D.F., Seitzinger S.P., Havens K.E., Lancelot C., Likens G.E. (2009). Controlling Eutrophication: Nitrogen and Phosphorus. Science.

[4] Voulvoulis N., Arpon K.D., Giakoumis T. (2017). The EU Water Framework Directive: From Great Expectations To Problems with Implementation. Sci. Total Environ..

[5] Kiriukhin M.Y., Collins K.D. (2002). Dynamic Hydration Numbers for Biologically Important Ions. Biophys. Chem..

[6] Cao Z., Gordiichuk P.I., Loos K., Sudholter E.J.R., de Smet L.C.P.M. (2016). The Effect of Guanidinium Functionalization on the Structural Properties and Anion Affinity of Polyelectrolyte Multilayers. Soft Matter.

[7] Kim M., Kim H., Byeon S.-H. (2017). Layered Yttrium Hydroxide l-Y(OH)3 Luminescent Adsorbent for Detection and Recovery of Phosphate from Water over a Wide pH Range. ACS Appl. Mater. Interfaces.

[8] Huang Y., Yang J.-K., Keller A.A. (2014). Removal of Arsenic and Phosphate from Aqueous Solution by Metal (Hydr-)oxide Coated Sand. ACS Sustainable Chem. Eng..

[9] Weng L., van Riemsdijk W.H., Hiemstra T. (2008). Humic Nanoparticles at the Oxide−Water Interface: Interactions with Phosphate Ion Adsorption. Environ. Sci. Technol..

[10] Karagollu O., Gorur M., Gode F., Sennik B., Yilmaz F. (2014). Phosphate ion sensors based on triazole connected ferrocene moieties. Sens. Actuators B.

[11] Paltrinieri L., Wang M., Sachdeva S., Besseling N.A.M., Sudholter E.J.R., de Smet L.C.P.M. (2017). Fe_3_O_4_ Nanoparticles Coated with a Guanidinium-functionalized Polyelectrolyte Extend the pH Range for Phosphate Binding. J. Mater. Chem. A.

[12] Alexander C., Andersson H.S., Andersson L.I., Ansell R.J., Kirsch N., Nicholls I.A., O’Mahony J., Whitcombe M.J. (2006). Molecular Imprinting Science and Technology: A Survey of the Literature for the Years up to and Including 2003. J. Mol. Recognit..

[13] Sibrian-Vazquez M., Spivak D.A. (2004). Molecular Imprinting Made Easy. J. Am. Chem. Soc..

[14] Chen L., Wang X., Lu W., Wu X., Li J. (2016). Molecular Imprinting: Perspectives and Applications. Chem. Soc. Rev..

[15] Pei Y., Fan F., Wang X., Feng W., Hou Y., Pei Z. (2017). Fabrication of Hypericin Imprinted Polymer Nanospheres via Thiol-Yne Click Reaction. Polymers.

[16] Viveiros R., Rebocho S., Casimiro T. (2018). Green Strategies for Molecularly Imprinted Polymer Development. Polymers.

[17] Zhang H., Ye L., Mosbach K. (2006). Non-covalent Molecular Imprinting with Emphasis on its Application in Separation and Drug Development. J. Mol. Recognit..

[18] Kempe M., Mosbach K. (1995). Molecular Imprinting Used for Chiral Separations. J. Chromatogr. A.

[19] Göçenoğlu Sarıkaya A., Osman B., Çam T., Denizli A. (2017). Molecularly Imprinted Surface Plasmon Resonance (SPR) Sensor for Uric Acid Determination. Sens. Actuators B.

[20] Ertürk G., Uzun L., Tümer M.A., Say R., Denizli A. (2011). Fab Fragments Imprinted SPR Biosensor for Real-time Human Immunoglobulin G Detection. Biosens. Bioelectron..

[21] Whitcombe M.J., Chianella I., Larcombe L., Piletsky S.A., Noble J., Porter R., Horgan A. (2011). The Rational Development of Molecularly Imprinted Polymer-based Sensors for Protein Detection. Chem. Soc. Rev..

[22] Rao T.P., Kala R., Daniel S. (2006). Metal Ion-imprinted Polymers—Novel Materials for Selective Recognition of Inorganics. Anal. Chim. Acta.

[23] Vidyasankar S., Arnold F.H. (1995). Molecular Imprinting: Selective Materials for Separations, Sensors and Catalysis. Curr. Opin. Biotechnol..

[24] Dickert F.L., Halikias K., Hayden O., Piu L., Sikorski R. (2001). Sensors Based on Fingerprints of Neutral and Ionic Analytes in Polymeric Materials. Sens. Actuators B.

[25] Hart B.R., Shea K.J. (2002). Molecular Imprinting for the Recognition of N-Terminal Histidine Peptides in Aqueous Solution. Macromolecules.

[26] Iskierko Z., Sharma P.S., Prochowicz D., Fronc K., D’Souza F., Toczydłowska D., Stefaniak F., Noworyta K. (2016). Molecularly Imprinted Polymer (MIP) Film with Improved Surface Area Developed by Using Metal–Organic Framework (MOF) for Sensitive Lipocalin (NGAL) Determination. ACS Appl. Mater. Interfaces.

[27] Poma A., Brahmbhatt H., Watts J.K., Turner N.W. (2014). Nucleoside-Tailored Molecularly Imprinted Polymeric Nanoparticles (MIP NPs). Macromolecules.

[28] Zhao Y., Ma Y., Li H., Wang L. (2012). Correction to Composite QDs@MIP Nanospheres for Specific Recognition and Direct Fluorescent Quantification of Pesticides in Aqueous Media. Anal. Chem..

[29] Chen Y., Li D., Bie Z., He X., Liu Z. (2016). Coupling of Phosphate-Imprinted Mesoporous Silica Nanoparticles-Based Selective Enrichment with Matrix-Assisted Laser Desorption Ionization-Time-of-Flight Mass Spectrometry for Highly Efficient Analysis of Protein Phosphorylation. Anal. Chem..

[30] Rimola A., Costa D., Sodupe M., Lambert J.-F., Ugliengo P. (2013). Silica Surface Features and Their Role in the Adsorption of Biomolecules: Computational Modeling and Experiments. Chem. Rev..

[31] Chibowski E., Szcześ A., Hołysz L. (2010). Changes of Zeta Potential and Particles Size of Silica Caused by DPPC Adsorption and Enzyme Phospholipase A2 Presence. Adsorption.

[32] Ma M., Zheng S., Chen H., Yao M., Zhang K., Jia X., Mou J., Xu H., Wu R., Shi J. (2014). A Combined “RAFT” and “Graft From” Polymerization Strategy for Surface Modification of Mesoporous Silica Nanoparticles: Towards Enhanced Tumor Accumulation and Cancer Therapy Efficacy. J. Mater. Chem. B.

[33] Lundqvist M., Sethson I., Jonsson B.-H. (2004). Protein Adsorption onto Silica Nanoparticles:  Conformational Changes Depend on the Particles’ Curvature and the Protein Stability. Langmuir.

[34] Rapuano R., Carmona-Ribeiro A.M. (1997). Physical Adsorption of Bilayer Membranes on Silica. J. Colloid Interface Sci..

[35] Cui J., Wang Y., Hao J., Caruso F. (2009). Mesoporous Silica-Templated Assembly of Luminescent Polyester Particles. Chem. Mater..

[36] Jun-Hwan P., Young-Ho L., Seong-Geun O. (2007). Preparation of Thermosensitive PNIPAm-Grafted Mesoporous Silica Particles. Macromol. Chem. Phys..

[37] Wu T., Zhang Y., Wang X., Liu S. (2008). Fabrication of Hybrid Silica Nanoparticles Densely Grafted with Thermoresponsive Poly(*N*-isopropylacrylamide) Brushes of Controlled Thickness via Surface-Initiated Atom Transfer Radical Polymerization. Chem. Mater..

[38] Liu C.-H., Pan C.-Y. (2007). Grafting Polystyrene onto Silica Nanoparticles via RAFT Polymerization. Polymer.

[39] Rapuano R., Carmona-Ribeiro A.M. (2000). Supported Bilayers on Silica. J. Colloid Interface Sci..

[40] Moura S.P., Carmona-Ribeiro A.M. (2005). Biomimetic Particles: Optimization of Phospholipid Bilayer Coverage on Silica and Colloid Stabilization. Langmuir.

[41] Tero R., Takizawa M., Li Y., Yamazaki M., Urisu T. (2004). Deposition of Phospholipid Layers on SiO_2_ Surface Modified by Alkyl-SAM Islands. Appl. Surf. Sci..

[42] Castellana E.T., Cremer P.S. (2006). Solid Supported Lipid Bilayers: From Biophysical Studies to Sensor Design. Surf. Sci. Rep..

[43] Troutier A.-L., Ladaviere C. (2007). An Overview of Lipid Membrane Supported by Colloidal Particles. Adv. Colloid Interface Sci..

[44] Liu Z.-J., Liang Y.-L., Geng F.-F., Lv F., Dai R.-J., Zhang Y.-K., Deng Y.-L. (2012). Preparation of Poly (*N*-isopropylacrylamide) Brush Grafted Silica Particles via Surface-initiated Atom Transfer Radical Polymerization Used for Aqueous Chromatography. Front. Mater. Sci..

[45] Schild H.G. (1992). Poly(*N*-isopropylacrylamide): Experiment, theory and application. Prog. Polym. Sci..

[46] Cheng J., Shan G., Pan P. (2015). Temperature and pH-dependent swelling and copper(ii) adsorption of poly(*N*-isopropylacrylamide) copolymer hydrogel. RSC Adv..

[47] Liu Y., Shen L. (2008). From Langmuir Kinetics to First-and Second-order Rate Equations for Adsorption. Langmuir.

[48] Federation W.E., Association A.P.H. (2005). Standard Methods for the Examination of Water and Wastewater.

[49] Chen Y., Li X., Yin D., Li D., Bie Z., Liu Z. (2015). Dual-template docking oriented molecular imprinting: A facile strategy for highly efficient imprinting within mesoporous materials. Chem. Commun..

[50] Zhang L., Zhou N., Wang B., Liu C., Zhu G. (2014). Fabrication of Fe_3_O_4_/PAH/PSS@ Pd Core–shell Microspheres by Layer-by-layer Assembly and Application in Catalysis. J. Colloid Interface Sci..

[51] Cao Z., Du B., Chen T., Nie J., Xu J., Fan Z. (2008). Preparation and Properties of Thermo-sensitive Organic/Inorganic Hybrid Microgels. Langmuir.

[52] Humphreys B.A., Willott J.D., Murdoch T.J., Webber G.B., Wanless E.J. (2016). Specific Ion Modulated Thermoresponse of Poly (*N*-isopropylacrylamide) Brushes. Phys. Chem. Chem. Phys..

