# Supplementary material for: Fabrication of Phosphate-Imprinted PNIPAM/SiO2 Hybrid Particles and Their Phosphate Binding Property
Source: Polymers (Basel). 2019 Feb 2;11(2):253. doi: 10.3390/polym11020253 (PMC6419039; doi:10.3390/polym11020253)
Supplement: Supplementary file 1 [file polymers-11-00253-s001.pdf]

Fabrication of Phosphate-imprinted PNIPAM/SiO_2 Hybrid Particles and Their Phosphate Binding Property

Zheng Cao[1, 2], Yuyuan Chen[1], Dan Li[1], Junfeng Cheng[1, 3, *], and Chunlin Liu[1, 4, *]

[1] Jiangsu Key Laboratory of Environmentally Friendly Polymeric Materials, School of Materials Science and Engineering, Jiangsu Collaborative Innovation Center of Photovoltaic Science and Engineering, Changzhou University, Changzhou, Jiangsu, 213164, China; zcao@cczu.edu.cn (Z.C.); 15189781265@163.com (Y.C.); 15151992892@163.com (D.L.);

[2]- Key Laboratory of Synthetic and Self-Assembly Chemistry for Organic Functional Molecules, Shanghai Institute of Organic Chemistry, Chinese Academy of Sciences, 345 Lingling Road, Shanghai 200032, China;

[3] National Experimental Demonstration Center for Materials Science and Engineering (Changzhou University), Changzhou, 213164, P.R. China;

[4] Huaide College, Changzhou University, Changzhou, 213016, P.R. China;

* Correspondence: junfeng@cczu.edu.cn (J.C.); chunlin@cczu.edu.cn (C.L.); Prof. Liu's Tel.: +86-0519-8633-0095

1 Prepare a standard curve

1.1 Preparation of ammonium molybdate solution

(1) Dissolve 9.57 g of ammonium molybdate tetrahydrate in 500 mL of water.

(2) Add 0.2 g of antimony potassium tartrate and 80 mL of concentrated sulfuric acid and cool down.

(3) Dilute to 1000 mL with water, and store in brown bottle.

1.2 Preparation of ascorbic acid solution

(1) Dissolve 17.6 g of ascorbic acid in about 500 mL of water.

(2) Dilute to 1000 mL with water, and store in a brown bottle.

1.3 Preparation of phosphate standard solution

(1) Dissolve 0.7165g of potassium dihydrogen phosphate and dilute to 1000 mL. 1 mL of this solution contained 0.5 mg of phosphate ions.

(2) Pipette 20 mL of the solution (1) and dilute to 500 mL. 1 mL of this solution contained 0.02 mg of phosphate ions.

1.4 Drawing of standard curve

(1) Take 1.0, 2.0, 3.0, 4.0, 5.0, 6.0, 7.0, and 8.0 mL of the standard solution (phosphate concentration: 0.02 mg/mL) in 8 sets of 50 mL volumetric flasks, and dilute with about 25 mL of water.

(2) Add 5.0 mL of Ammonium molybdate solution, 3.0 mL of ascorbic acid solution to each solution (1), and diluted to 50 mL with water, and allowed to stand at room temperature for 10 minutes.

(3) The absorbance was measured with a cuvette and a reagent blank. Prepare a standard curve by plotting the absorbance values of standards at 890 nm versus the corresponding phosphate concentrations.

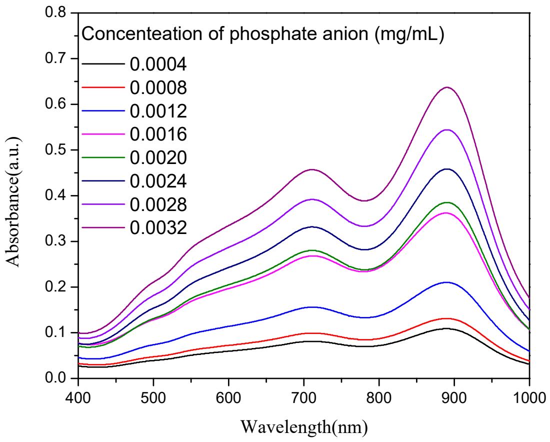

Figure S1. UV-vis spectra of the phosphate standard solutions.

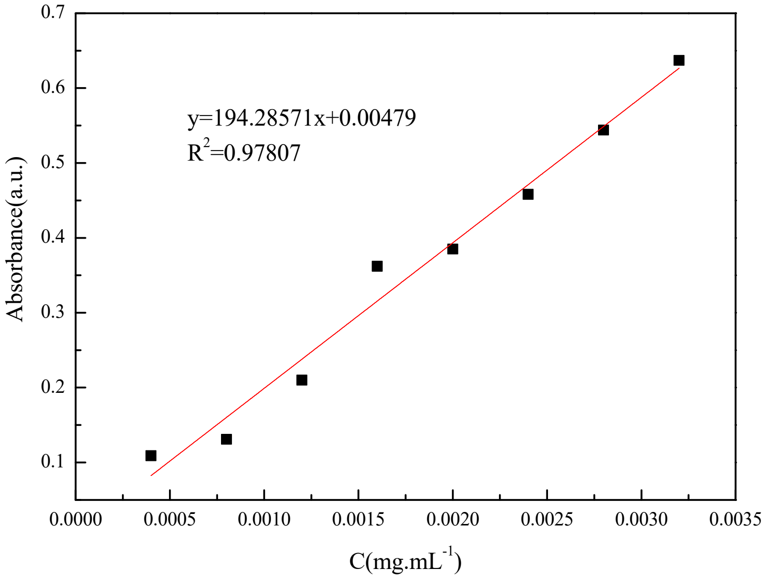

Figure S2. A standard curve (Absorbance vs Phosphate concentration).

2 SEM and TEM images of phosphate imprinted SiO_2 and PNIPAM/SiO_2 microspheres

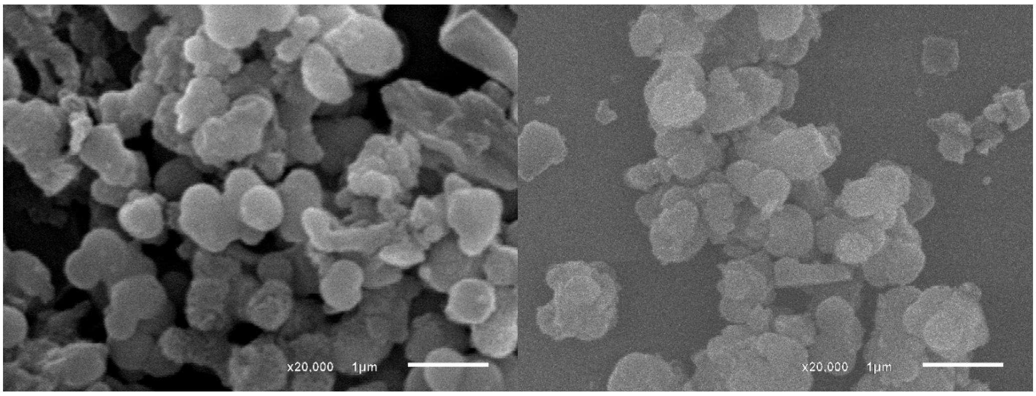

Figure S3. SEM images of phosphate imprinted mesoporous SiO_2 (Left) and PNIPAM/SiO_2 (Right).

It can be seen from the SEM image of phosphate-imprinted mesoporous silica particles that has a spherical shape with a particle size of about 500 nm, some of the particles are aggregated. The SEM image from PNIPAM/SiO_2 also exhibits a spherical shape, and some particles are aggregated, and the particle size is about 500 nm, which is consistent with the particle size distribution test results.

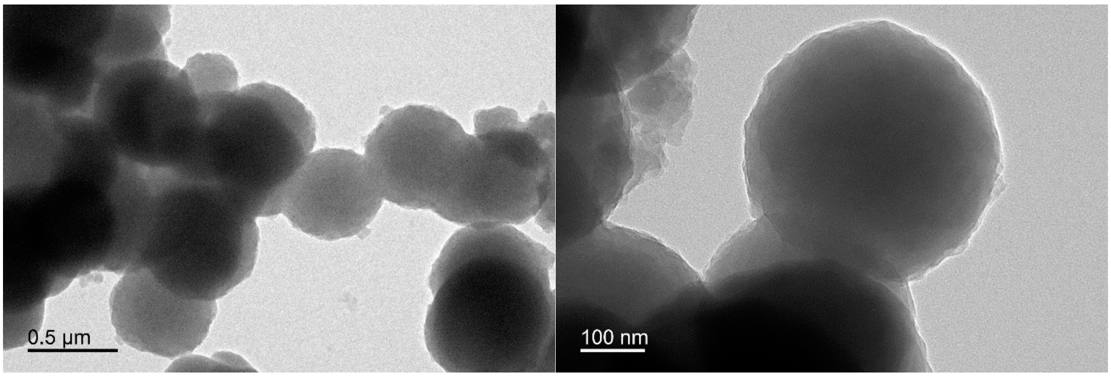

Figure S4. TEM images of PNIPAM/SiO_2 hybrid particles (a 0.5 μm , b 100 nm).

3 The size and size distribution of the phosphate imprinted SiO_2 particles dispersed in water.

The laser particle size analyser was used to determine the size and size distribution of the phosphate imprinted SiO_2 particles dispersed in water. Figure S5 shows the size distribution of the phosphate imprinted SiO_2 particles dispersed in water at pH = 7 and T = 25 °C .

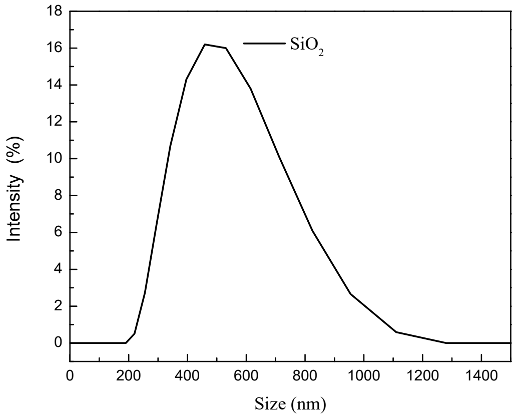

Figure S5. The size distribution of the phosphate imprinted SiO_2 microspheres.

3 Preparation and characterization of PNIPAM microgels

3.1 Preparation of PNIPAM microgel

0.891 g of NIPAM (N-isopropylacrylamide, 99%, J&K Scientific Ltd., Beijing, China) was dissolved in 43 mL of deionized water, then 225 μL of TMEDA (N,N,N',N'-Tetramethylethylenediamine, 99%, J&K Scientific Ltd., Beijing, China) and 0.18 g of MBA (N,N'-methylene bisacrylamide, 98%, J & K Scientific Ltd., Beijing, China) as cross-linker was added and mixed. After 10 min, 2 ml of $K_2S_2O_8$ solution (5 mg/mL) was added into the solution to initiate the polymerization. Oxygen was eliminated by bubbling nitrogen through the solution. The

reaction was continued at 70 °C for 6 hours. The microgels were then purified by extensive dialysis against deionized water. Water was changed every 12 hours for two days.

3.2 PNIPAM particle size

The particle size of PNIPAM microgels in water at 25 °C is about 518 nm.

3.3 SEM image of PNIPAM microgels

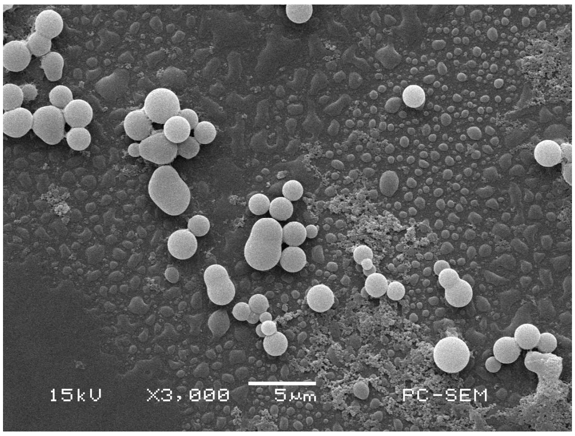

Figure S6. SEM image of PNIPAM microgels.

4 Phosphate adsorption capacity of PNIPAM/solid SiO_2 particles

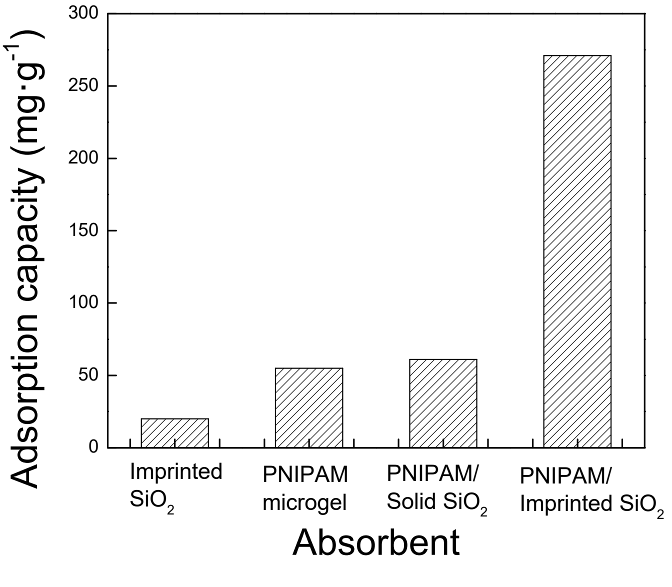

Figure S7. The comparison of phosphate adsorption capacity of phosphate imprinted SiO_2, PNIPAM microgels, PNIPAM@solid SiO_2, and PNIPAM@imprinted SiO_2.
